# Dose-response mapping of bladder and rectum in prostate cancer patients undergoing radiotherapy with and without baseline toxicity correction

**DOI:** 10.1016/j.phro.2025.100805

**Published:** 2025-07-01

**Authors:** Tanuj Puri, Tiziana Rancati, Petra Seibold, Adam Webb, Eliana Vasquez Osorio, Andrew Green, Eliana Gioscio, David Azria, Marie-Pierre Farcy-Jacquet, Jenny Chang-Claude, Alison Dunning, Maarten Lambrecht, Barbara Avuzzi, Dirk de Ruysscher, Elena Sperk, Ana Vega, Liv Veldeman, Barry Rosenstein, Jane Shortall, Sarah Kerns, Christopher Talbot, Andrew P. Morris, Alan McWilliam, Peter Hoskin, Ananya Choudhury, Catharine West, Marcel van Herk, REQUITE Consortium

**Affiliations:** a Division of Cancer Sciences, The Christie NHS Foundation Trust, The University of Manchester, UK; b Data Science Unit, Fondazione IRCCS Istituto Nazionale dei Tumori, Milan, Italy; c Division of Cancer Epidemiology, German Cancer Research Center (DKFZ), Heidelberg, Germany; d University of Leicester, UK; e EMBL’s European Bioinformatics Institute, Cambridge, UK; f L’Institut du Cancer de Montpellier, Department of Radiation Oncology, Montpellier, France; g Centre Hospitalier Régional Universitaire de Nîmes, Nimes, France; h University of Cambridge, UK; i Department of Oncology, KU Leuven, Belgium; j Unit of radiotherapy 1, Fondazione IRCCS Istituto Nazionale dei Tumori, Milan, Italy; k Maastricht University Medical Center, School for Oncology and Developmental Biology Grow, Maastricht, Netherlands; l Department of Radiation Oncology, Universitätsmedizin Mannheim, Medical Faculty Mannheim, Germany; m Grupo Genética en Cáncer y Enfermedades Raras, Instituto de Investigación Sanitaria de Santiago de Compostela, Santiago de Compostela, Spain, and, Fundación Pública Galega de Medicina Xenómica, Santiago de Compostela, Spain; n Ghent University Hospital, Ghent, Belgium; o Mount Sinai Hospital, New York, USA; p Medical College of Wisconsin, Milwaukee, USA; q Division of Musculoskeletal & Dermatological Sciences, The University of Manchester, UK

**Keywords:** Prostate cancer, Radiotherapy, VBA, IBDM, Dose-toxicity modeling, Organ-at-risk

## Abstract

**Background and purpose:**

Radiotherapy dose–response maps (DRM) combine dose-surface maps (DSM) and toxicity outcomes to identify high-risk subregions in organ-at-risk. This study assesses the impact of baseline toxicity correction on the identification of high-risk subregions in dose–response modeling for prostate cancer patients undergoing radiotherapy.

**Materials and methods:**

The analysis included 1808 datasets, with 589 exclusions before toxicity-specific data removal. Bladder/rectum were automatically segmented on planning computed tomography scans, DSMs unwrapped into 91x90 voxel grids, and converted to equivalent doses in 2 Gy fractions (EQD2; α/β = 1 Gy). Seventeen late toxicities were assessed with two methods: (i) baseline toxicity subtracted from the maximum of 12- and 24-months toxicity scores, dichotomized at grade 1, and (ii) maximum of 12- and 24-months toxicity scores dichotomized at grade 1. DSMs were split accordingly, and voxel-wise t-values computed using Welch’s t-equation. Statistically significant voxels were identified via the 95th percentile of maximum of t-value (Tmax) distribution.

**Results:**

Event counts with baseline correction were 82/82/286/226 for urinary tract obstruction/retention/urgency/incontinence, respectively; without baseline correction, they were 93/104/465/361. For bladder DSMs, urinary incontinence, obstruction, retention, and urgency had 1143/186, 1768/1848, 516/0, and 33/0 significant voxels without/with baseline correction. For rectum DSMs, urinary incontinence and tract obstruction had 604/0 and 1980/889 significant voxels without/with baseline correction. However, no significant associations between rectal DSMs and rectum-related toxicities were found.

**Conclusions:**

DRM without baseline correction appears more sensitive to high-risk subregions due to higher event counts. Non-linear toxicity grading and multivariable analysis may enhance DRM reliability.

## 1 Introduction

Prostate cancer (PCa) affects approximately 52000 individuals/year in the UK [1], with around 30% receiving curative radiotherapy [2]. External beam radiotherapy targets tumors but requires expanded radiation margins to account for organ motion, inadvertently exposing adjacent organ-at-risk (OAR), such as bladder/rectum. This increases the risk of radiation-induced toxicities, such as rectal bleeding and urinary obstruction, which can impact patients' quality-of-life.

Dose-volume histograms (DVH) [3] are commonly used to assess dose-toxicity relationships but rely on simplifying assumptions: uniform dose distribution, tissue homogeneity, uniform tissue response, and independent volume effects. These limitations hinder their ability to capture complex OAR responses. To address this, voxel-based dose–response mapping (DRM) [[4], [5], [6], [7], [8]] has emerged, correlating two-dimensional (2D, for hollow organs) or three-dimensional (3D, for solid organs) dose distributions—such as dose-surface maps (DSM)—with toxicity outcomes. This approach helps identify high-risk subregions within OARs, potentially guiding treatment optimization. However, DRM results are influenced by model parameters and the number of observed toxicity events [9,10]. A key unresolved issue is whether methodological choices, such as baseline correction, affect the identification of high-risk subregions.

Univariable image-based data mining (IBDM)/voxel-based analysis (VBA), which uses dose as the sole predictor for the toxicity outcome, has been widely applied across various cancer types [[4], [5], [6], [7], [8]]. However, univariable approaches fail to account for confounding factors, such as baseline toxicity, which can increase the likelihood of genitourinary complications in PCa patients [11]. This is particularly relevant for urinary obstruction, as its occurrence within two years of radiotherapy is linked to pre-existing urinary dysfunction, acute toxicity, prior transurethral prostate resection, and localized bladder dose hotspots. As previous studies have not corrected for baseline toxicity, we explicitly applied this correction prior to including toxicity data in the dose-toxicity prediction model—an approach not previously explored in the literature—to better isolate radiation-induced effects.

This study compares two methods of incorporating late toxicities—with and without baseline correction—within univariable VBA/IBDM to assess their impact on identifying high-risk bladder and rectal subregions in PCa radiotherapy patients.

## 2 Material and methods

Data from 1808 prostate cancer patients in the multinational REQUITE trial (2014–2017) were included [12], with 589 unique exclusions prior to toxicity-specific filtering. Radiotherapy was administered per local protocols. Informed patient consent was obtained. All procedures complied with relevant regulations and received ethics approval (UK: North West − GM East REC, ref: 14/NW/0035). The study is registered at ISRCTN98496463.

Patient demographics, characteristics and treatment summary were assessed. Thereafter, due to large variation in manual contouring, the bladder/rectum contours were re-segmented automatically on planning computed tomography (CT) scans using a commercial software (ADMIRE, Elekta, Sweden) [13,14] and used for the DRM analysis presented throughout this manuscript. Mid-axial slices of segmented organs were saved as JPEGs for visual review.

Dose data were resampled via trilinear interpolation to match CT voxel resolutions (X/Y: 0.85–2.71 mm; Z: 1–5 mm).

Bladder DSMs were generated via spherical unwrapping (radius *r*, θ: 0–180° generating 91 sampling points in 2°/θ intervals, φ: 0–360° generating 90 sampling points in 4°/φ intervals, using WorldMatch software [15,16]). Spherical unwrapping utilized the bladder’s center of mass. Unwrapping errors occurred near the bladder trigone were visually identified and excluded.

Rectal DSMs used cylindrical coordinates (radius *r*, θ: 0–360°, height *z*). The cylindrical DSMs were generated via posterior contour incision, unwrapped posterior-medial to distal-right and anterior-medial to distal-left, centering the high-dose region. Irregular contours (e.g., crescents) occasionally displaced slice centers outside the contour, causing unwrapping artifacts; dose values were linearly interpolated from adjacent slices where feasible.

DSMs were spatially normalized to a 91 × 90 voxel grids (trilinear interpolation) for cross-patient comparability accounting for differences in bladder/rectum sizes among all patients. To account for differences in treatment fractionation, dose values were converted to equivalent doses in 2 Gy fractions (EQD2) using α/β = 1 [[17], [18], [19], [20], [21], [22]].

Seventeen late toxicities (Common Terminology Criteria for Adverse Events (CTCAE) clinician-graded 0–3 at baseline, 12- or 24-months) included proctitis, perforation, bowel obstruction, fistula, bowel stenosis, bowel ulceration, diarrhoea, flatus, rectal bleeding, management of sphincter control, hematuria, urinary tract obstruction, urinary incontinence, urinary frequency, urinary urgency, urinary retention and bowel fistula. The 24-month data reflect prevalence information rather than new incidence [12].

Two toxicity utilization methods were compared: (1) Baseline corrected: The maximum toxicity score at 12 and 24 months was adjusted by subtracting the baseline toxicity score. The resulting adjusted toxicity values were then divided into two groups: if the adjusted value was 1 or higher (grade ≥ 1), it was classified as the event group (assigned a value of 1); if it was below 1, it was classified as the non-event group (assigned a value of 0). This method assumes that (i) the relationship between DSMs and post-radiotherapy toxicities is solely due to radiation-induced damage, excluding baseline toxicity pre-radiotherapy, and (ii) toxicity severity grades follow a linear scale, with equal clinical impact between consecutive grades (e.g., grade 3 vs. grade 2, grade 1 vs. grade 0); and (2) Non-baseline corrected: The maximum toxicity score at 12 and 24 months was taken without adjustment. This score was then divided into two groups: if the score was 1 or higher (grade ≥ 1), it was classified as the event group (assigned a value of 1); if it was below 1, it was classified as the non-event group (assigned a value of 0). This method assumes a non-linear toxicity scale, where differences between higher grades (e.g., grade 3 vs. grade 2) are more clinically significant than those between lower grades (e.g., grade 2 vs. grade 1), potentially better reflecting clinical reality.

Data normalcy at 8190 voxel locations per group was assessed using the Shapiro-Wilk test. Voxel-wise t-values were then calculated using Welch’s t-equation to compare DSMs between the event and non-event groups [23], accounting for unequal variances and sample sizes: t=(X_1_−X_2_)/sqrt((S_1_^2^/N_1_)+(S_2_^2^/N_2_)), where X_1_, X_2_ are group means; S_1_^2^, S_2_^2^ are variances; N_1_, N_2_ are sample sizes. This yielded a 2D map of 8190 t-values reflecting voxel-wise group differences.

**Fig. 1:**
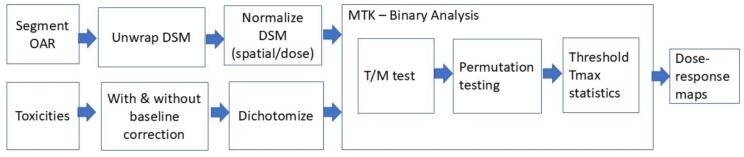
Dose-response mapping (DRMs) methodology. Arrows describe the direction of operation. Compressed data received in ‘.tar.gz’ file format was unzipped with a bash script, and DICOM-formatted data were segmented using ADMIRE software accessed via a batch script, with results saved as DICOM file format. A PowerShell script was used to rename unzipped files, exclude empty folders, and verify and count folders with data present in them. Dose, CT and contours datasets were converted to the local PACK format using WorldMatch software (developed using C/C++/Delphi) accessed via a Lua script. Dose-surface mapping, spatial normalization and EQD2 conversion were performed in WorldMatch via Lua scripting in extensible markup language (XML) data reduced schema (XDR) file format. Dose-response mapping that included statistical analysis and permutation testing were obtained using the MTK (developed using C/C++) accessed through a Python wrapper. The DRM visualisation, contour plotting, voxel counting, and DSC calculation was performed in Python. ADMIRE = Advanced Modeled Iterative Reconstruction; CT = Computed Tomography; DSC = Dice Similarity Coefficient; DSM = Dose-Surface Maps; DICOM = Digital Imaging and Communications in Medicine; EQD2 = Equivalent Dose in 2 Gy Fractions; MTK = Manchester Tool Kit; OAR = Organs-at-Risk; QA = Quality Assurance; T/M = Welch t equation.

Permutation testing (1000 iterations [23]) established significance thresholds via the 95th percentile of the ordered maximum t-values (Tmax), correcting for multiple comparisons. Fig. 1 shows the DRM methodology.

Overlap between DRMs from the two methods (without/with baseline correction) were quantified via Dice similarity coefficient (DSC) [24,25].

See Supplemental sections S1 for manual contouring variation, S2 for DSM unwrapping, S3 for DSC, S4 for EQD2 and S5 for data exclusion details.

## 3 Results

Patient demographics, characteristics and treatment details (mean ± standard deviation) were: age 69.3 ± 7.1 yrs, height 172.6 ± 12.8 cm, weight 82.3 ± 14.6 kg, prescribed dose 71.4 ± 6.7 Gy, fractions 33.8 ± 6.7, dose/fraction 2.2 ± 0.4 Gy, planning tumor volume 138.9 ± 69.8 cm^3^, automatically segmented bladder volume 162.9 ± 123.8 cm^3^, rectum volume 68.4 ± 23.5 cm^3^ and rectum length 9.7 ± 1.3 cm (along anterior-posterior axis). Clinical T-stage distribution was: T1a (0.3%), T1b (0.0%), T1c (22.6%), T2a/b (14.1% each), T2c (10.8%), T3a (11.2%), T3b (3.0%), T4 (1.3%), and unknown (21.75%).

Of the 17 toxicities analyzed, only statistically significant ones are reported: urinary tract obstruction, retention, urgency, and incontinence. Without baseline correction, counts were 93, 104, 465, and 361 (rates: 8%, 9%, 40%, and 31%). With baseline correction, event counts were 82, 82, 286, and 226 (rates: 7%, 7%, 25%, and 19%).

Despite non-normalcy in all non-event group voxels and approximately 25% of event group voxels (based on Shapiro-Wilk test), Welch’s t-equation was used.

**Fig. 2:**
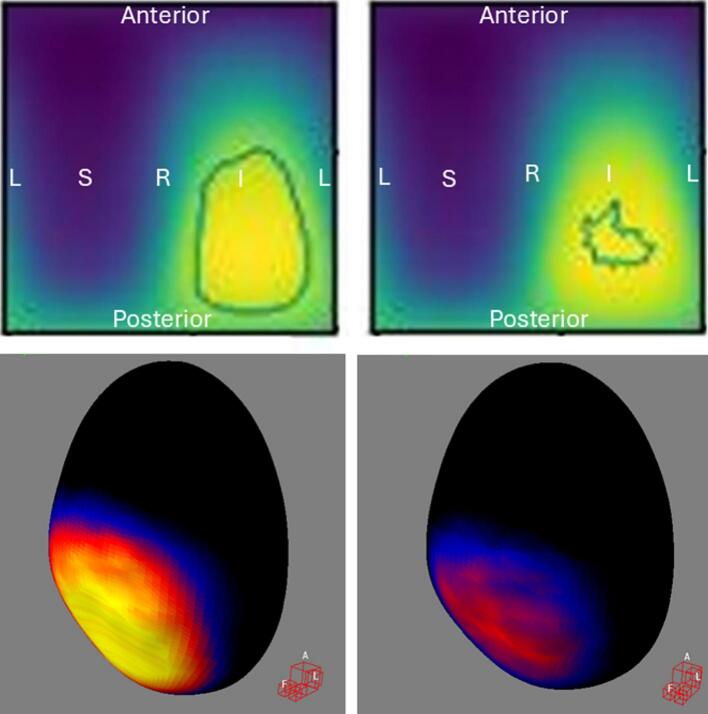
Baseline toxicity subtraction reduced statistically significant high-risk subregions in high-dose voxels for **urinary incontinence**. In the top row, the left figure shows **bladder** DRM without baseline correction (mean DSM with minimum/maximum value: 18/69 Gy, 361 events), while the right shows DRM with baseline correction (mean DSM with minimum/maximum value: 18/69 Gy, 226 events). Statistically significant voxels within contours: 1143/186 without/with baseline correction (DSC = 0.28). Figures result from spherical unwrapping (α/β = 1, 91x90 voxel grid, Welch t-equation, 1000 iterations, 95th percentile). Colors indicate mean dose: yellow (highest) to black (lowest). DRM = Dose–Response Map, DSM = Dose-Surface Map, DSC = Dice Similarity Coefficient, L=Patient’s left, R=Patient’s right, S=Patient’s superior, I=Patient’s inferior. In the bottom row, the left and right figures show the 3D t-maps without and with baseline correction, corresponding to the images in the top row. The Siddon view at the bottom-right corner of each 3D rendering indicates the patient’s anatomical orientation, with A, L, and F corresponding to anterior, left, and feet positions. Colors represent t-values from Welch’s *t*-equation: yellow (highest), through red and blue, to black (lowest). The colored regions correspond to the locations of the high-risk subregions outlined as contours in the top row. (For interpretation of the references to colour in this figure legend, the reader is referred to the web version of this article.)

**Fig. 3:**
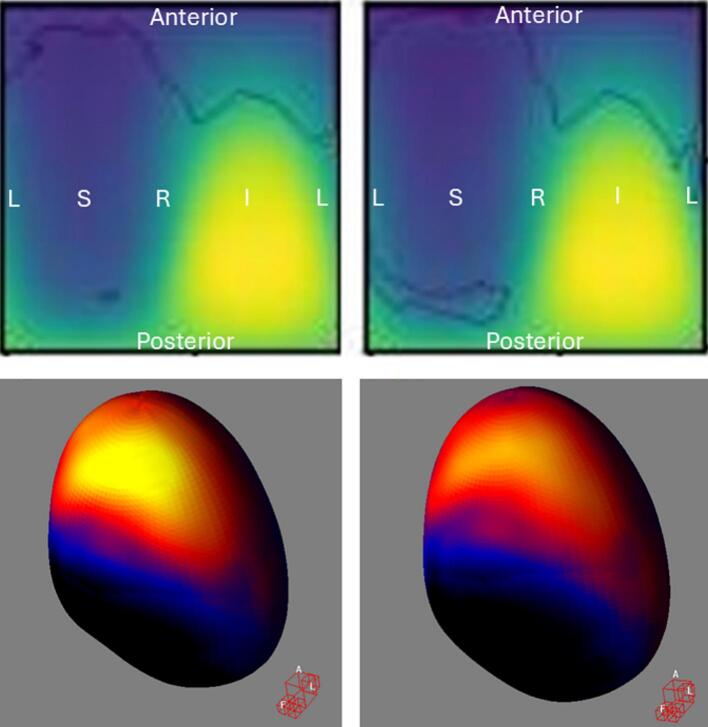
Baseline toxicity subtraction increased statistically significant high-risk subregions in low-dose voxels for **urinary tract obstruction**. In the top row, the left figure shows **bladder** DRM without baseline correction (mean DSM with minimum/maximum value: 18/69 Gy, 93 events), while the right shows DRM with baseline correction (mean DSM with minimum/maximum value: 18/69 Gy, 82 events). Statistically significant voxels within contours: 1768/1748 without/with baseline correction (DSC = 0.82). Contour is closed from the image boundaries, suggesting that the smaller region represents a higher risk, which is also evident from the number of statistically significant voxels in this 8190-voxel image. Figures result from spherical unwrapping (α/β = 1, 91x90 voxel grid, Welch t-equation, 1000 iterations, 95th percentile). Colors indicate mean dose: yellow (highest) to black (lowest). DRM = Dose–Response Map, DSM = Dose-Surface Map, DSC = Dice Similarity Coefficient, L=Patient’s left, R=Patient’s right, S=Patient’s superior, I=Patient’s inferior. In the bottom row, the left and right figures show the 3D t-maps without and with baseline correction, corresponding to the images in the top row. The Siddon view at the bottom-right corner of each 3D rendering indicates the patient’s anatomical orientation, with A, L, and F corresponding to anterior, left, and feet positions. Colors represent t-values from Welch’s *t*-equation: yellow (highest), through red and blue, to black (lowest). The colored regions correspond to the locations of the high-risk subregions outlined as contours in the top row. (For interpretation of the references to colour in this figure legend, the reader is referred to the web version of this article.)

**Fig. 4:**
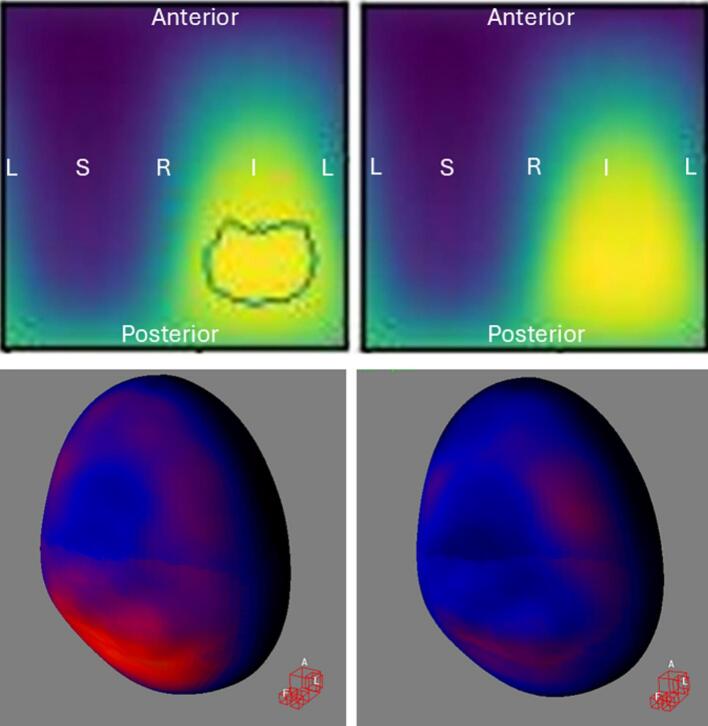
Baseline toxicity subtraction reduced statistically significant high-risk subregions in high-dose voxels for **urinary retention.** In the top row, the left figure shows **bladder** DRM without baseline correction (mean DSM with minimum/maximum value: 18/69 Gy, 104 events), while the right shows DRM with baseline correction (mean DSM with minimum/maximum value: 18/69 Gy, 82 events). Statistically significant voxels within contours: 516/0 without/with baseline correction (DSC = 0.0). Figures result from spherical unwrapping (α/β = 1, 91x90 voxel grid, Welch t-equation, 1000 iterations, 95th percentile). Colors indicate mean dose: yellow (highest) to black (lowest). DRM = Dose–Response Map, DSM = Dose-Surface Map, DSC = Dice Similarity Coefficient, L=Patient’s left, R=Patient’s right, S=Patient’s superior, I=Patient’s inferior. In the bottom row, the left and right figures show the 3D t-maps without and with baseline correction, corresponding to the images in the top row. The Siddon view at the bottom-right corner of each 3D rendering indicates the patient’s anatomical orientation, with A, L, and F corresponding to anterior, left, and feet positions. Colors represent t-values from Welch’s *t*-equation: yellow (highest), through red and blue, to black (lowest). The colored regions correspond to the locations of the high-risk subregions outlined as contours in the top row. (For interpretation of the references to colour in this figure legend, the reader is referred to the web version of this article.)

**Fig. 5:**
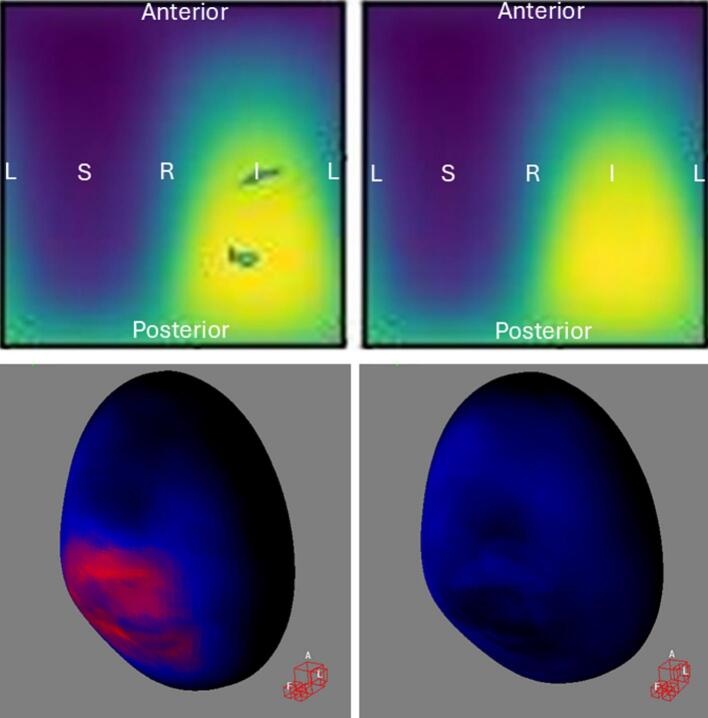
Baseline toxicity subtraction reduced statistically significant high-risk subregions in high-dose voxels for **urinary urgency**. In the top row, the left figure shows **bladder** DRM without baseline correction (mean DSM with minimum/maximum value: 18/69 Gy, 465 events), while the right shows DRM with baseline correction (mean DSM with minimum/maximum value: 18/69 Gy, 286 events). Statistically significant voxels within contours: 33/0 without/with baseline correction (DSC = 0.0). Figures result from spherical unwrapping (α/β = 1, 91x90 voxel grid, Welch t-equation, 1000 iterations, 95th percentile). Colors indicate mean dose: yellow (highest) to black (lowest). DRM = Dose–Response Map, DSM = Dose-Surface Map, DSC = Dice Similarity Coefficient, L=Patient’s left, R=Patient’s right, S=Patient’s superior, I=Patient’s inferior. In the bottom row, the left and right figures show the 3D t-maps without and with baseline correction, corresponding to the images in the top row. The Siddon view at the bottom-right corner of each 3D rendering indicates the patient’s anatomical orientation, with A, L, and F corresponding to anterior, left, and feet positions. Colors represent t-values from Welch’s *t*-equation: yellow (highest), through red and blue, to black (lowest). The colored regions correspond to the locations of the high-risk subregions outlined as contours in the top row. (For interpretation of the references to colour in this figure legend, the reader is referred to the web version of this article.)

For bladder DSMs, urinary incontinence (Fig. 2) and urinary tract obstruction (Fig. 3) showed significant DRM subregions without amd with baseline correction, while urinary retention (Fig. 4) and urgency (Fig. 5) were significant only without baseline correction. Statistically significant voxels without/with baseline correction: incontinence (1143/186, DSC = 0.28, in high-dose region), obstruction (1768/1848, DSC = 0.82, in low-dose region), retention (516/0, DSC = 0.0, in high-dose region), and urgency (33/0, DSC = 0.0, in high-dose region).

**Fig. 6:**
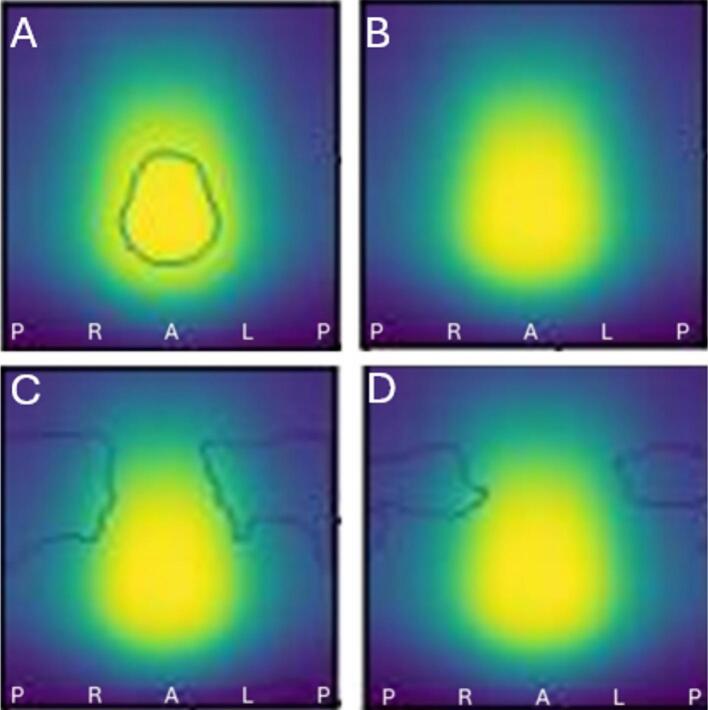
(A, B) Baseline toxicity subtraction reduced statistically significant high-risk subregions in high-dose voxels for **urinary incontinence.** The left figure (A) shows **rectum** DRM without baseline correction (mean DSM with minimum/maximum value: 4/74 Gy, 361 events), while the right (B) shows DRM with baseline correction (mean DSM with minimum/maximum value: 4/74 Gy, 226 events). Significant voxels within contours: 604/0 without/with baseline correction (DSC = 0.0). (C, D) Baseline toxicity subtraction reduced statistically significant high-risk subregions in low-dose voxels for **urinary tract obstruction**. The left figure (C) shows **rectum** DRM without baseline correction (mean DSM with minimum/maximum value: 4/74 Gy, 93 events), while the right (D) shows DRM with baseline correction (mean DSM with minimum/maximum value: 4/74 Gy, 82 events). Statistically significant voxels within contours: 1980/889 without/with baseline correction (DSC = 0.62). Contour is closed from the image boundaries, suggesting that the smaller region represents a higher risk, which is also evident from the number of statistically significant voxels in this 8190-voxel image. Figures (A-D) result from cylindrical unwrapping (α/β = 1, 91x90 voxel grid, Welch t-equation, 1000 iterations, 95th percentile). Colors indicate mean dose: yellow (highest) to black (lowest). DRM = Dose–Response Map, DSM = Dose-Surface Map, DSC = Dice Similarity Coefficient, L=Patient’s left, R=Patient’s right, P=Patient’s posterior, A=Patient’s anterior. (For interpretation of the references to colour in this figure legend, the reader is referred to the web version of this article.)

For rectum DSMs, urinary incontinence (Fig. 6**A-B**) and urinary tract obstruction (Fig. 6**C-D**) showed significant DRM subregions. Significant voxels without/with baseline correction: incontinence (604/0, DSC = 0.0, in high-dose region), obstruction (1980/889, DSC = 0.62, in low-dose region). Rectum DSMs showed no association with rectum-related toxicities.

Bladder (Fig. 2, Fig. 3) and rectum DRMs (Fig. 6**C-D**) showed approximately concentric significant subregions without and with baseline correction, differing in size.

## 4 Discussion

This study compares two univariable VBA/IBDM approaches for late toxicity analysis: one without baseline correction and one with. Many dose-response maps for the bladder and rectum showed concentric subregions of statistical significance, with more high-risk voxels identified in the uncorrected analysis—likely due to a higher number of events. While the uncorrected approach may appear more sensitive, baseline correction may reduce false positives by accounting for pre-treatment toxicity. However, as no univariable VBA/IBDM approach has been clinically validated, it remains unclear whether a higher voxel count overestimates risk due to lower statistical thresholds, or whether a lower voxel count underestimates risk by increasing thresholds and potentially excluding true positives.

Urinary incontinence and obstruction exhibited significant subregions in both bladder and rectum DRMs, likely due to combined radiation damage to both organs. This may be attributed to the high correlation between bladder and rectum doses in areas where the two organs are anatomically close. In contrast, retention and urgency were significant only in the bladder DRM, potentially due to false positives in rectum DRMs caused by non-tissue-specific genetic mutations. Additionally, discrepancies between planned and delivered doses to the rectum, influenced by its proximity to the prostate and the relatively low event rates, may further explain these differences [26]. Additionally, other uncertainties, such as the accuracy of cylindrical/spherical geometric mapping, may have also influenced the DSM results, potentially impacting the accuracy of the DRM.

Previous studies have explored the relationship between late urinary toxicities and bladder DSMs in PCa patients undergoing radiotherapy. Yahya et al. [27] found a significant association between radiation dose to the posteroinferior bladder, particularly the trigone, and late urinary incontinence, which aligns with our findings (Fig. 2). They also identified subregions with negative coefficients, indicating that higher doses correlated with lower toxicity risk, consistent with our results (Fig. 3). Mylona et al. [28,29] found an association between doses to the posterosuperior bladder and late urinary retention, which slightly differs from our results (Fig. 4). Gioscio et al. [30] observed significant associations between high-dose posterior bladder and late urinary frequency, and both high- and low-dose posterior subregions for urinary urgency, but no association with urinary incontinence. In contrast, our study did not find significant subregions for urinary frequency (Fig. 5), though very small high-risk subregions for urinary urgency were identified in high dose posteroinferior bladder areas. We did not find significant subregions in the rectum DSM for late rectal toxicities. Shelley et al. [26] used finite element modeling to create rectum DSMs and found significant associations with rectal bleeding, proctitis, stool frequency, and fecal incontinence. Onjukka et al. [31], applying Chen et al.'s technique [32], identified significant links between rectum DSMs and fecal incontinence and rectal bleeding. These discrepancies may stem from methodological differences, including sample size, toxicity grading, DSM methods, α/β value, spatial normalization, and statistical approaches, complicating direct comparisons and causing variations in results. Standardizing VBA/IBDM techniques for dose-toxicity analysis would improve comparability across centers.

Genetic mutations may influence early and late toxicities, with non-tissue-specific and tissue-specific effects, respectively [[33], [34], [35], [36]]. Determining whether baseline toxicities are tissue-specific is crucial, as their inclusion in radiation dose-based models could enhance late toxicity predictions. In the absence of genetic data, baseline correction can help isolate radiation-induced effects, improving prediction accuracy. Incorporating baseline toxicities in dose-toxicity models, such as multivariable VBA/IBDM, would further refine accuracy by accounting for baseline toxicity, clinical and genetic predispositions to radiotherapy resistance [37]. While baseline correction simplifies analysis, it remains essential when genetic or pre-existing factors cannot be explicitly considered [[33], [34], [35], [36]].

While 3D VBA/IBDM captures spatial dose-toxicity correlations, it is computationally intensive for hollow organs (such as bladder/rectum) due to redundant analysis of non-contributing doses (e.g., air/excreta). 2D DSMs simplify analysis by mapping surface doses, preserving spatial patterns where radiation-induced toxicities originate. This study used DSMs as a practical 2D approximation of 3D dose distribution, balancing efficiency with clinical relevance.

Bladder DRMs for urinary retention and urgency in Fig. 4, Fig. 5 showed significant subregions only without baseline correction, suggesting the findings are unlikely false positives due to the large number of events. The lack of significance with baseline correction may reflect reduced event count, indicating that some toxicities require more events for detection. Consistency between Fig. 2, Fig. 4, Fig. 5, where significant subregions align with high-dose areas, supports the dose-toxicity relationship. The differences between methods likely stem from event count, but the minimum event threshold for significance and the point at which increasing events no longer change results require further investigation.

Using α/β = 1 is justified for urinary toxicities [20,22]. Most studies conventionally use α/β≈3 for rectal tissue. However, our rationale for selecting α/β = 1 was methodological rather than clinical, aimed at enhancing sensitivity of the model. The EQD2 model is inherently non-linear and becomes progressively less sensitive to changes in fraction size as α/β increases. For instance, for a dose of 69 Gy delivered in 32 fractions (2.16 Gy/fraction), EQD2 decreases from 71.70 Gy at α/β = 2, to 70.54 Gy at α/β = 5, and further to 69.90 Gy at α/β = 10. This reduction in EQD2 spread at higher α/β values leads to lower variance across voxel-level dose distributions across patients, reducing the statistical power of detecting dose–response relationships. We previously found that increasing α/β consistently reduced the number of statistically significant high-risk voxels on OARs [10]. Even at α/β = 1, which should theoretically maximise sensitivity due to greater dose variance, no significant associations between rectal DSMs and rectal toxicities were found, suggesting that applying α/β = 3—where EQD2 values would vary even less—is unlikely to yield different results. Thus, the choice of α/β = 1 was intended to explore the upper bound of model sensitivity, not to introduce bias.

Despite Shapiro-Wilk test failures at most voxel locations, Welch’s t-equation was used for two reasons: (i) The Central Limit Theorem asserts that large sample sizes (≥30) lead to normal distributions for the sum or mean of independent variables, justifying Welch's t-equation despite voxel correlation violating independence assumption. This correlation may bias results by exaggerating significance, suggesting that methods like mixed-effects models or cluster-based permutation tests may be more appropriate. (ii) The nonparametric Mann-Whitney U-equation failed to identify significant voxels in the DRM for any of the 17 toxicities analyzed.

The test statistic, Tmax, represents the most extreme of 8190 voxel-wise t-values and was assessed using 1000 permutations, where outcome labels were randomly flipped and DSMs reshuffled into event and non-event groups for each permutation. The 95th percentile of the resulting permutation distribution was assigned a P-value of 0.05, marking the top 5% of Tmax values [32]. We selected permutation testing as it remains the most widely used method in VBA/IBDM studies [32,38] and offers a balanced approach—less conservative than Bonferroni correction [39], which can overly reduce statistical power and increase Type II errors (false negatives), but more conservative than the Benjamini–Hochberg method [40], which may increase the risk of Type I errors (false positives). As a data-driven method well-suited to high-dimensional analyses, permutation testing offers robust control of both Type I and Type II errors. In the absence of standardized and validated frameworks within the evolving VBA/IBDM field, adopting widely accepted methodologies was essential to ensure the reliability of our findings.

A critical question remains: are univariable parametric or non-parametric frameworks relying solely on dose data sufficient to identify high-risk OAR subregions for treatment planning? Univariable VBA/IBDM approaches—using Welch’s t or Mann–Whitney U equations—have key limitations: (i) These methods remain unvalidated across cancer types, limiting their reliability in identifying high-risk subregions or predicting outcomes. They assess group differences but are insensitive to uniform dose scaling—statistical significance can persist despite changes in toxicity risk. For example, doubling dose (e.g., 60 → 120 Gy) significantly increases toxicity, yet univariable models may yield unchanged results, lacking biological plausibility and weakening their application for risk stratification. (ii) Rancati et al. [41] showed that incorporating genomic data into VBA/IBDM for prostate cancer shifted identified high-risk bladder subregions from low- to high-dose areas, highlighting the limitations of dose-only-based models. This supports the need for multivariable VBA/IBDM that integrates planning dose, baseline toxicity, genomics, and clinical/biological factors to improve accuracy. Therefore, univariable dose-only VBA/IBDM lacks clinical utility unless combined with clinical/genomic variables in multivariable models, because, first, it cannot adjust for confounders, explaining paradoxical findings in prior studies [10,26,27,30,31,[42], [43], [44], [45], [46], [47]], where event groups received lower mean EQD2 in high-risk subregions—contradicting expectations. It remains unclear if negative voxel values were truncated or absent. For instance, Witte et al. [44] reported significant dose differences outside prostate/seminal vesicle targets, with non-failure groups receiving higher doses in presacral and obturatorial regions, while failure groups exhibited negative dose differences. Such inconsistencies reflect univariable methods’ failure to capture spatial and biological complexities, producing results misaligned with clinical logic. Second, our study uniquely assesses how baseline toxicity correction affects DRM results when dose is the sole predictor—unexplored in prior work—offering a technical, not clinical, framework.

Future REQUITE analyses by treatment center may reveal center-specific anomalies. Modality-based stratification (e.g., volumetric-modulated arc therapy versus intensity-modulated radiation) could identify distribution confounders. Causal modeling could identify/adjust for confounders to estimate true cause-effect relationships. Also, merging radical and post-prostatectomy (salvage/adjuvant) cohort is discouraged due to differing treatment intents and prostatectomy-induced neural changes, which may heighten sexual toxicity risks linked to rectum/bladder OARs—warranting cohort-specific analysis.

In conclusion, without or with baseline toxicity correction methods impact high-risk subregion identification in bladder/rectum in univariable dose–response modeling in PCa patients undergoing radiotherapy. While baseline uncorrected methods appear more sensitive, baseline correction reduces false positives by accounting for pre-treatment symptoms. To accurately assess radiation-induced late toxicities, baseline data must be incorporated either pre-emptively or post hoc. Given the non-linearity of toxicity grades, alternative correction strategies should be explored. For reproducibility and external comparison, future VBA/IBDM studies should report methodology detail, including toxicity modeling and event counts by toxicity type, and restrict DSM analysis to tissue-specific toxicities (e.g., bladder endpoints for bladder DRMs, not rectum DRMs). Ultimately, integrating baseline toxicity, genomics, clinical variables, and technical factors into multivariable (not univariable) models may enhance the predictive power of toxicity prediction frameworks.

## CRediT authorship contribution statement

Conceptualization: TP. Data curation: TR, PS, AW, DA, MPFJ, JCC, AD, ML, BA, DDR, EP, AV, LV, BR, SK, CT, PH, AC, CW, MVH, REQUITE Consortium members and staff in 26 hospitals across 8 countries. Formal analysis: TP. Methodology: TP, TR, PS, EVO, EG, DA, MPFJ, JCC, AD, ML, BA, DDR, EP, AV, LV, BR, AMW, PH, AC, CW, MVH. Resources: AMW, MVH. Software: TP, EVO, AG, MVH. Supervision: MVH. Visualization: TP, MVH. Writing - original draft: TP. Writing - review & editing: TP, TR, PS, AW, EVO, AG, EG, DA, MPFJ, JCC, AD, ML, BA, DDR, ES, AV, LV, BR, JS, SK, CT, APM, AMW, PH, AC, CW, MVH.

## Declaration of competing interest

This work was funded by Movember, Prostate Cancer UK (RIA15-ST2-031), and The Christie NHS Foundation Trust. Marcel van Herk and Ananya Choudhury were supported by the National Institute for Health and Care Research (NIHR) Manchester Biomedical Research Centre (NIHR203308). Catharine West would like to acknowledge BRC/RADNET. This research was made possible through funding from the European Union - Seventh Framework Programme for Research, Technological Development, and Demonstration, under Grant ID: 601826. Ana Vega: supported by Spanish Instituto de Salud Carlos III (ISCIII) funding, an initiative of the Spanish Ministry of Economy and Innovation partially supported by European Regional Development FEDER Funds (PI22/00589, PI19/01424; INT24/00023); the ERAPerMed JTC2018 funding (AC18/00117); the Autonomous Government of Galicia (Consolidation and structuring program: IN607B), and by the AECC (PRYES211091VEGA). Dirk de Ruysscher obtained grants and/or contracts from (i) AstraZeneca/BMS/Beigene/Philips/Olink: Research grant/support/Advisory Board: Institutional financial interests (no personal financial interests), and (ii) Eli-Lilly: Advisory Board: Institutional financial interests (no personal financial interests). All other authors declare no competing interests. The University of Manchester (UoM) has covered the cost of open access publication associated with this manuscript as UoM is a Jisc member with Elsevier.

## References

[1] Department of Health and Social Care, The Rt Hon Victoria Atkins MP. Biggest prostate cancer screening trial in decades to start in UK. Published 19 November 2023. https://www.gov.uk/government/news/biggest-prostate-cancer-screening-trial-in-decades-to-start-in-uk ; [accessed 21 June 2024].

[2] Cancer treatments: Find out more about treatment data for patients who have received chemotherapy, radiotherapy and surgical tumour resections for their tumour in England. https://digital.nhs.uk/ndrs/data/data-outputs/cancer-data-hub/cancer-treatments; [accessed 21 June 2024].

[3] Gty C. (1988). Dose volume histograms in treatment planning. Int J Radiat Oncol Biol Phys.

[4] Acosta O., Drean G., Ospina J.D., Simon A., Haigron P., Lafond C. (2013). Voxel-based population analysis for correlating local dose and rectal toxicity in prostate cancer radiotherapy. Phys Med Biol.

[5] Palma G., Monti S., Cella L. (2020). Voxel-based analysis in radiation oncology: a methodological cookbook. Phys Med.

[6] McWilliam A., Kennedy J., Hodgson C., Vasquez Osorio E., Faivre-Finn C., Van Herk M. (2017). Radiation dose to heart base linked with poorer survival in lung cancer patients. Eur J Cancer.

[7] Vasquez Osorio E., Abravan A., Green A., Van Herk M., Lee L.W., Ganderton D. (2023). Dysphagia at 1 year is associated with mean dose to the inferior section of the brain stem. Int J Radiat Oncol.

[8] Palma G., Monti S., D’Avino V., Conson M., Liuzzi R., Pressello M.C. (2016). A voxel-based approach to explore local dose differences associated with radiation-induced lung damage. Int J Radiat Oncol.

[9] Puri T., van Herk M., Vasquez Osorio E., Shortall J., Morris A., Kerns S. (2023 Jul 23–27). 65th Annual Meeting & Exhibition, AAPM.

[10] Puri T., van Herk M., Vasquez Osorio E., Morros A.P., Shortall J., Kerns S.L. (2024). Sensitivity of dose–response mapping model parameters at the bladder and rectum in prostate cancer. Radiother Oncol.

[11] Kim S., Moore D.F., Shih W., Lin Y., Li H., Shao Y.-H. (2013). Severe genitourinary toxicity following radiation therapy for prostate cancer—how long does it last?. J Urol.

[12] Seibold P., Webb A., Aguado-Barrera M.E., Azria D., Bourgier C., Brengues M. (2019). REQUITE: a prospective multicentre cohort study of patients undergoing radiotherapy for breast, lung or prostate cancer. Radiother Oncol.

[13] O’Connell M. Automatic image segmentation using deep convolutional neural networks – a white paper. C3 P1 Confidential and Proprietary © Elekta Group; 2020.

[14] Bordigoni B., Trivellato S., Pellegrini R., Meregalli S., Bonetto E., Belmonte M. (2024). Automated segmentation in pelvic radiotherapy: a comprehensive evaluation of ATLAS-, machine learning-, and deep learning-based models. Phys Med.

[15] Van Herk M, De Jaeger K, De Munck J, Hoogeman M, Meinders J, Ploeger L, et al. A delineation system for N modalities — software aspects. In: Schlegel W, Bortfeld T, editors. Use Comput. Radiat. Ther., Berlin, Heidelberg: Springer Berlin Heidelberg; 2000, p. 73–5. https://doi.org/10.1007/978-3-642-59758-9_27.

[16] Remeijer P., Rasch C., Lebesque J.V., Van Herk M. (1999). A general methodology for three‐dimensional analysis of variation in target volume delineation. Med Phys.

[17] McMahon S.J. (2018). The linear quadratic model: usage, interpretation and challenges. Phys Med Biol.

[18] Brand D.H., Brüningk S.C., Wilkins A., Fernandez K., Naismith O., Gao A. (2021). Estimates of alpha/beta (α/β) ratios for individual late rectal toxicity endpoints: an analysis of the CHHiP trial. Int J Radiat Oncol.

[19] Brand D.H., Brüningk S.C., Wilkins A., Naismith O., Gao A., Syndikus I. (2023). The fraction size sensitivity of late genitourinary toxicity: analysis of alpha/beta (α/β) ratios in the CHHiP trial. Int J Radiat Oncol.

[20] Fiorino C., Cozzarini C., Rancati T., Briganti A., Cattaneo G.M., Mangili P. (2014). Modelling the impact of fractionation on late urinary toxicity after postprostatectomy radiation therapy. Int J Radiat Oncol.

[21] Cui M., Gao X.-S., Li X., Ma M., Qi X., Shibamoto Y. (2022). Variability of α/β ratios for prostate cancer with the fractionation schedule: caution against using the linear-quadratic model for hypofractionated radiotherapy. Radiat Oncol.

[22] Dörr W., Hendry J.H. (2001). Consequential late effects in normal tissues. Radiother Oncol.

[23] Ebert M.A., Gulliford S., Acosta O., De Crevoisier R., McNutt T., Heemsbergen W.D. (2021). Spatial descriptions of radiotherapy dose: normal tissue complication models and statistical associations. Phys Med Biol.

[24] Dice L. (1945). Measures of the amount of ecologic association between species. Ecology.

[25] Sørensen T. (1948). A method of establishing groups of equal amplitude in plant sociology based on similarity of species and its application to analyses of the vegetation on danish commons. K Dan Vidensk Selsk.

[26] Shelley L.E.A., Sutcliffe M.P.F., Thomas S.J., Noble D.J., Romanchikova M., Harrison K. (2020). Associations between voxel-level accumulated dose and rectal toxicity in prostate radiotherapy. Phys Imaging Radiat Oncol.

[27] Yahya N., Ebert M.A., House M.J., Kennedy A., Matthews J., Joseph D.J. (2017). Modeling urinary dysfunction after external beam radiation therapy of the prostate using bladder dose-surface maps: evidence of spatially variable response of the bladder surface. Int J Radiat Oncol.

[28] Mylona E., Cicchetti A., Rancati T., Palorini F., Fiorino C., Supiot S. (2020). Local dose analysis to predict acute and late urinary toxicities after prostate cancer radiotherapy: Assessment of cohort and method effects. Radiother Oncol.

[29] Mylona E., Acosta O., Lizee T., Lafond C., Crehange G., Magné N. (2019). Voxel-based analysis for identification of urethrovesical subregions predicting urinary toxicity after prostate cancer radiation therapy. Int J Radiat Oncol.

[30] Gioscio E., Cicchetti A., Iacovacci J., Spampinato S., Waskiewicz J.M., Avuzzi B. (2023). PO-2099 Bladder dose surface maps identify subregions associated to late toxicities after prostate cancer RT. Radiother Oncol.

[31] Onjukka E., Fiorino C., Cicchetti A., Palorini F., Improta I., Gagliardi G. (2019). Patterns in ano-rectal dose maps and the risk of late toxicity after prostate IMRT. Acta Oncol.

[32] Chen C., Witte M., Heemsbergen W., Herk M.V. (2013). Multiple comparisons permutation test for image based data mining in radiotherapy. Radiat Oncol.

[33] Barnett G.C., Thompson D., Fachal L., Kerns S., Talbot C., Elliott R.M. (2014). A genome wide association study (GWAS) providing evidence of an association between common genetic variants and late radiotherapy toxicity. Radiother Oncol.

[34] Naderi E., Aguado-Barrera M.E., Schack L.M.H., Dorling L., Rattay T., Fachal L. (2023). Large-scale meta–genome-wide association study reveals common genetic factors linked to radiation-induced acute toxicities across cancer types. JNCI Cancer Spectr.

[35] Kishan A.U., Marco N., Schulz-Jaavall M.-B., Steinberg M.L., Tran P.T., Juarez J.E. (2022). Germline variants disrupting microRNAs predict long-term genitourinary toxicity after prostate cancer radiation. Radiother Oncol.

[36] Benitez C.M., Knox S.J. (2020). Harnessing genome-wide association studies to minimize adverse radiation-induced side effects. Radiat Oncol J.

[37] Gioscio E., Massi M.C., Franco N.R., Seibold P., Avuzzi B., Cicchetti A. (2024). 1378: Genetically-based analysis of dose surface maps for assessing toxicity post-RT: an innovative method. Radiother Oncol.

[38] Noguchi K., Konietschke F., Marmolejo-Ramos F., Pauly M. (2021). Permutation tests are robust and powerful at 0.5% and 5% significance levels. Behav Res Methods.

[39] Dewey ME, Seneta E. Carlo Emilio Bonferroni. In: Heyde CC, Seneta E, Crépel P, Fienberg SE, Gani J, editors. Stat. Centuries, New York, NY: Springer New York; 2001, p. 411–4. https://doi.org/10.1007/978-1-4613-0179-0_88.

[40] Benjamini Y., Hochberg Y. (1995). Controlling the False Discovery Rate: a Practical and Powerful Approach to Multiple Testing. J R Stat Soc Ser B Stat Methodol.

[41] Rancati T. Dose-response mapping at the bladder in prostate cancer patients undergoing radiotherapy: Success, challenges, and future direction. Presented at: RadioGenomics Consortium Annual Meeting; 2024 Oct 10–11; Aarhus University Hospital, Aarhus, Denmark.

[42] Puri T, Gioscio E, van Herk M, Seibold P, Vasquez Osorio E, et al. Comparison of two independently developed voxel-based dose-response mapping algorithms at two different centres for bladder and rectum in prostate cancer patients undergoing radical radiotherapy. 66th American Association of Physicists in Medicine (AAPM); 2024. Los Angeles, USA.

[43] Witte M., Pos F., Incrocci L., Heemsbergen W. (2021). Association between incidental dose outside the prostate and tumor control after modern image-guided radiotherapy. Phys Imaging Radiat Oncol.

[44] Witte M.G., Heemsbergen W.D., Bohoslavsky R., Pos F.J., Al-Mamgani A., Lebesque J.V. (2010). Relating dose outside the prostate with freedom from failure in the dutch trial 68 Gy vs. 78 Gy. Int. J Radiat Oncol.

[45] Wortel R.C., Witte M.G., Van Der Heide U.A., Pos F.J., Lebesque J.V., Van Herk M. (2015). Dose–surface maps identifying local dose–effects for acute gastrointestinal toxicity after radiotherapy for prostate cancer. Radiother Oncol.

[46] Improta I., Palorini F., Cozzarini C., Rancati T., Avuzzi B., Franco P. (2016). Bladder spatial-dose descriptors correlate with acute urinary toxicity after radiation therapy for prostate cancer. Phys Med.

[47] Palorini F., Cozzarini C., Gianolini S., Botti A., Carillo V., Iotti C. (2016). First application of a pixel-wise analysis on bladder dose–surface maps in prostate cancer radiotherapy. Radiother Oncol.

