# Supplementary material for: Dose-response mapping of bladder and rectum in prostate cancer patients undergoing radiotherapy with and without baseline toxicity correction
Source: Phys Imaging Radiat Oncol. 2025 Jul 1;35:100805. doi: 10.1016/j.phro.2025.100805 (PMC12272478; doi:10.1016/j.phro.2025.100805)
Supplement: Supplementary Data 1 [file mmc1.docx]

**SUPPLEMENTAL SECTIONS**

**Section S1:** Manual contouring

Manual delineations of the bladder were made on the entire bladder, on posterior wall only, or only a part of the bladder. Manual delineations of the rectum were made on entire rectum as solid organ (rectal wall and filling) from anus to sigmoid, or entire rectum as hollow organ (only rectal wall) from anus to sigmoid, or 2 cm above and below planning tumor volume (PTV) as solid organ, or 2 cm above and below PTV as hollow organ, or only a part of the rectum.

**Section S2:** Dose-surface mapping of bladder using spherical and rectum using cylindrical coordinates

To visualize dose distributions on the bladder surface, the three-dimensional (3D) dose data were transformed and unwrapped into a two-dimensional (2D) map using spherical coordinate parameterization. Cartesian coordinates (x,y,z) were first converted to spherical coordinates (r,θ,ϕ) using the transformations: r=sqrt(x^2^+y^2^+z^2^), θ=arccos(z/r), and ϕ=arctan(y/x), where r is the radial distance from the origin (bladder’s center of mass), θ is the polar angle measured from the superior-inferior axis, and ϕ is the azimuthal angle measured around the central axis. The bladder surface was assumed to be a sphere with a fixed radius r=R. The 3D bladder surface was then parameterized as a 2D map using u=ϕ(0≤u<2π) and v=θ(0≤v≤π), where u represents the azimuthal angle ϕ, corresponding to the circumferential position around the bladder, and v corresponds to the polar angle θ, representing the superior-inferior position. This transformation effectively unrolls the spherical bladder surface into a rectangular 2D representation, allowing for clearer visualization and analysis of dose distributions across different regions of the bladder wall.

To visualize dose distributions on the rectum surface, the 3D dose data were transformed and unwrapped into a 2D map using cylindrical coordinate parameterization. Cartesian coordinates (x,y,z) were first converted to cylindrical coordinates (r,ϕ,z) using the transformations: r=sqrt(x^2^+y^2^), ϕ=arctan(y/x), and z= z, where r is the radial distance from the center, ϕ is the azimuthal angle around the central axis, and z represents the height along the rectum’s longitudinal axis. The rectum surface was assumed to be at a fixed radius r=R, with a total height H along its central axis, extending from the superior end (z=0) to the inferior end (z=H). The 3D rectum surface was then parameterized as a 2D map using u = ϕ(0≤u<2π) and v=z(0≤v≤ H), where u represents the azimuthal angle ϕ around the rectum’s central axis, and v corresponds to the scaled height along the rectum’s central axis, determined from imaging data. This transformation effectively unrolls the cylindrical rectum surface into a rectangular 2D representation, allowing for clearer visualization and analysis of dose distributions across different regions of the rectum wall.

**Section S3:** Dice Similarity Coefficient (DSC)

DSC was calculated between the high-risk subregion contours identified with and without baseline toxicity correction. The DSC, defined as 2×∣A∩B∣/(∣A∣+∣B∣), measures similarity between sets A and B. Here, ∣A∣ and ∣B∣ denote the sizes of sets A and B, and ∣A∩B∣ is the size of their intersection. The coefficient ranges from 0 (no overlap) to 1 (complete overlap).

**Section S4:** Equivalent Doses in 2 Gy Fractions (EQD2)

To account for differences in treatment fractionation, dose values were converted to EQD2 using α/β=1, ensuring direct comparability across different treatment regimens. The linear-quadratic formula was used as EQD2=D*((d+α/β)/(2+α/β)), where D is the total dose, d is the dose per fraction (d=D/n), and α/β represents the dose at which the linear (α) and quadratic (β) effects on cell killing are equal. To calculate EQD2 for an arbitrary schedule with n fractions of dose d, the dose yielding an equal effect level is determined, where D=n*d.

**Section S5:** Data exclusions

Datasets were excluded for the following reasons: missing baseline, 12- or 24-month toxicity values (varied by toxicity type and method used with or without baseline correction so they were not counted); baseline toxicity exceeding post-treatment values (varied by toxicity type so they were not counted); empty data folders (N=150); visually incorrect rectum or bladder segmentations, failed or visually incorrect DSM (N=425); brachytherapy treatment (N=182; only volumetric-modulated arc therapy (VMAT), intensity-modulated radiation therapy (IMRT), or 3D conformal radiotherapy data included); missing prescribed dose (N=80); patients treated in the prone position (N=3). Overlaps existed among exclusions, e.g., data was checked for missing toxicity values regardless of organ-at-risk contouring success or failure. Total unique exclusions were 589, excluding the toxicity-specific exclusions depending on the method used with or without baseline correction.
